# Incidental finding of a giant intracardiac angioma infiltrating both ventricles in a 35-year-old woman: a case report

**DOI:** 10.1186/s13256-016-0860-4

**Published:** 2016-04-12

**Authors:** K. Hirschberg, F. Wiedmann, E. Zitron, P. Fortner, J. H. Riffel, E. Chorianopoulos, G. Gdynia, G. Mechtersheimer, M. Andrassy, G. Szabó, R. Arif, H. A. Katus, S. J. Buss

**Affiliations:** 1 Department of Cardiology, University of Heidelberg, Im Neuenheimer Feld 410, 69120 Heidelberg, Germany; 2 Institute of Pathology, University of Heidelberg, Im Neuenheimer Feld 220/221, 69120 Heidelberg, Germany; 3 Fürst-Stirum-Klinik, Gutleutstraße 1-14, 76646 Bruchsal, Germany; 4 Department of Cardiac Surgery, University of Heidelberg, Heidelberg, Germany

**Keywords:** CMR, Cardiac tumor, Echocardiography, Intracardiac angioma

## Abstract

**Background:**

Primary cardiac tumors are rare and often asymptomatic or present with unspecific symptoms. Benign cardiac tumors of vascular origin are especially rare, with only few existing data in the literature.

**Case presentation:**

A 35-year-old Caucasian female patient presented to our department with an asymptomatic giant intracardiac angioma infiltrating both ventricles. Evaluation of this tumor involved electrocardiography, echocardiography, cardiac magnetic resonance imaging, coronary angiography, an open myocardial biopsy, and histological examination of the resected specimen. Because our patient was asymptomatic, she was managed conservatively with regular follow-up. We discuss the treatment options available in comparison with similar cases.

**Conclusion:**

Diagnosis and therapy of benign cardiac tumors, especially of asymptomatic lesions, can be a challenge. There is no evidence available to help in the management of such patients. An extensive evaluation is needed with different imaging modalities, and case-specific decisions should be made that involve experts in cardiology, cardio-oncology, and heart surgery.

**Electronic supplementary material:**

The online version of this article (doi:10.1186/s13256-016-0860-4) contains supplementary material, which is available to authorized users.

## Background

Primary cardiac tumors are rare and their incidence ranges from 0.0017 to 0.28 % as reported in autopsy studies [1]. The most common benign tumors of the heart are myxomas, followed by lipoma, papillary fibroelastoma, angioma, fibroma, hemangioma, rhabdomyoma, and teratoma. Only about 5 % of all benign cardiac tumors are angiomas [2], and diagnosing them is often difficult. Many of the primary cardiac tumors are asymptomatic and are detected postmortem. If these tumors are symptomatic, embolization, obstruction, and arrhythmogenesis are the major modes of presentation [2].

## Case presentation

**Fig. 1:**
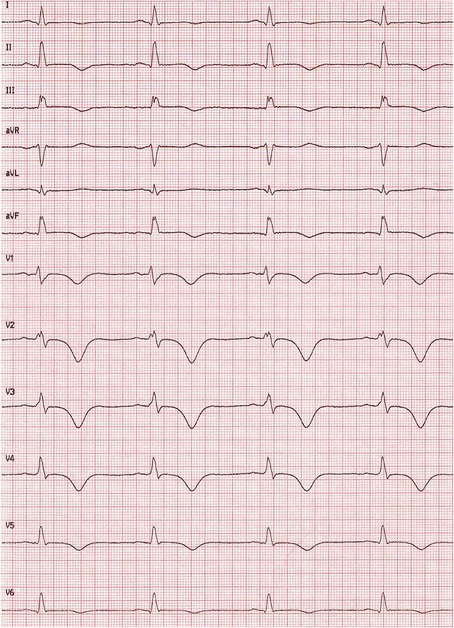
Results from a 12-lead electrocardiogram of the patient showing T-wave inversion in the inferior and precordial leads

**Fig. 2:**
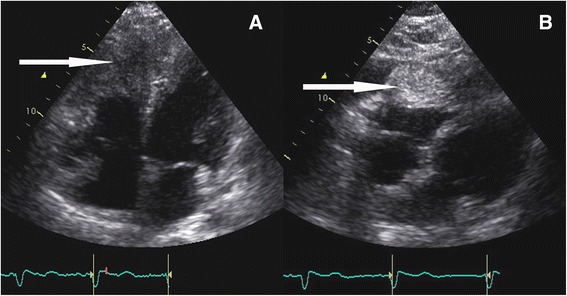
Apical four-chamber view (**a**) and subcostal four-chamber view (**b**) echocardiogram showing a large and homogenous mass in the right ventricle and pericardial effusion

**Fig. 3:**
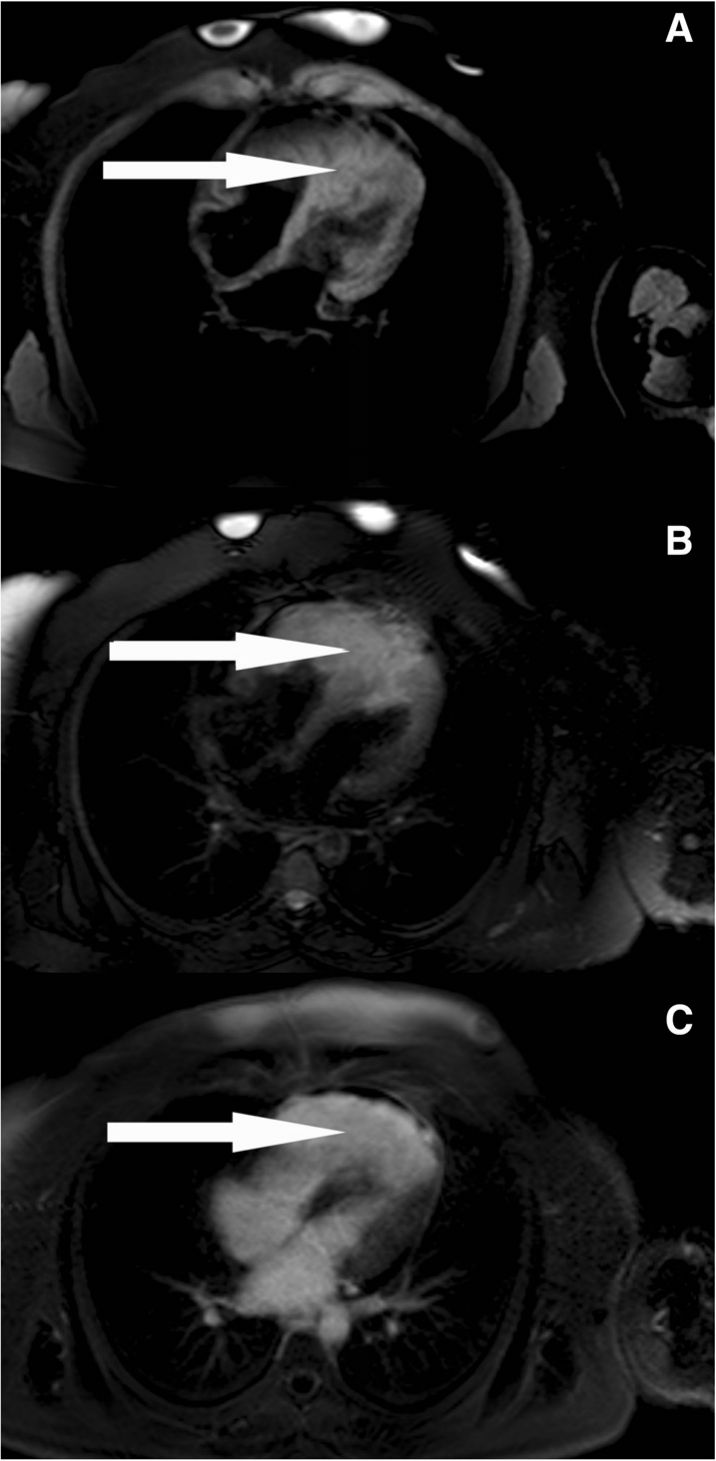
T1-weighted (**a**) and T2-weighted (**b**) images and late gadolinium enhancement (**c**) on cardiovascular magnetic resonance imaging

A 35-year-old Caucasian female patient was referred to our hospital because of an incidental finding of a large right ventricular mass during sonography of her upper abdominal organs performed for the evaluation of transient and moderate abdominal pain. Our patient did not have any specific cardiac symptoms like chest pain, dizziness, nausea, palpitations, syncope, or signs of congestive heart failure. Cardiovascular risk factors involved current smoking and obesity (body mass index 32.8 kg/m^2^). Her medical history included bronchial asthma, previous gestational diabetes, and minor depression. She was taking beclometasondipropionat, fluticason-17-propionat and formoterol-fumarate-dihydrate for the bronchial asthma, and fluoxetine for the depression. A cardiac murmur was not detected during a routine physical examination. Laboratory parameters were unremarkable, with no elevation in her levels of high-sensitive cardiac troponin T (7 pg/ml, reference <14 pg/ml), n-terminal pro-brain natriuretic peptide (75 ng/l, reference <125 ng/l), or C-reactive protein (4.5 mg/l, reference <5 mg/l). A 12-lead electrocardiogram showed T-wave inversion in the inferior and precordial leads (Fig. 1). Holter monitoring showed a normofrequent sinus rhythm without any supraventricular or ventricular ectopic beat. An exercise test revealed a good exercise capacity without chest pain, shortness of breath, or any other symptoms upon reaching a maximal heart rate of 163 beats per minute (93 % of the target heart rate). Her blood pressure and heart rate profile during exercise testing were normal and no ectopic beats were detected. Transthoracic echocardiography revealed a large homogenous mass in her slightly dilated right ventricle, suggesting the involvement of her intraventricular septum and left ventricular apex. Her cardiac valves were normal without stenosis or regurgitation, and the size and function of her left ventricle were normal. A small, not significant pericardial effusion was also detected (Fig. 2). Our patient then underwent cardiovascular magnetic resonance (CMR) imaging, which revealed a 104 × 62 mm right ventricular mass infiltrating her intraventricular septum and left ventricular apex. T1-weighted images showed isointensity and T2-weighted images showed clear hyperintensity of the relatively homogenous tumor (Fig. 3a, b). Late gadolinium enhancement depicted the dimensions of the tumor (Fig. 3c). Online supplemental video files show good systolic left ventricular function, slightly reduced right ventricular longitudinal function, and an intensive perfusion of the tumor (Additional files 1, 2 and 3).

**Fig. 4:**
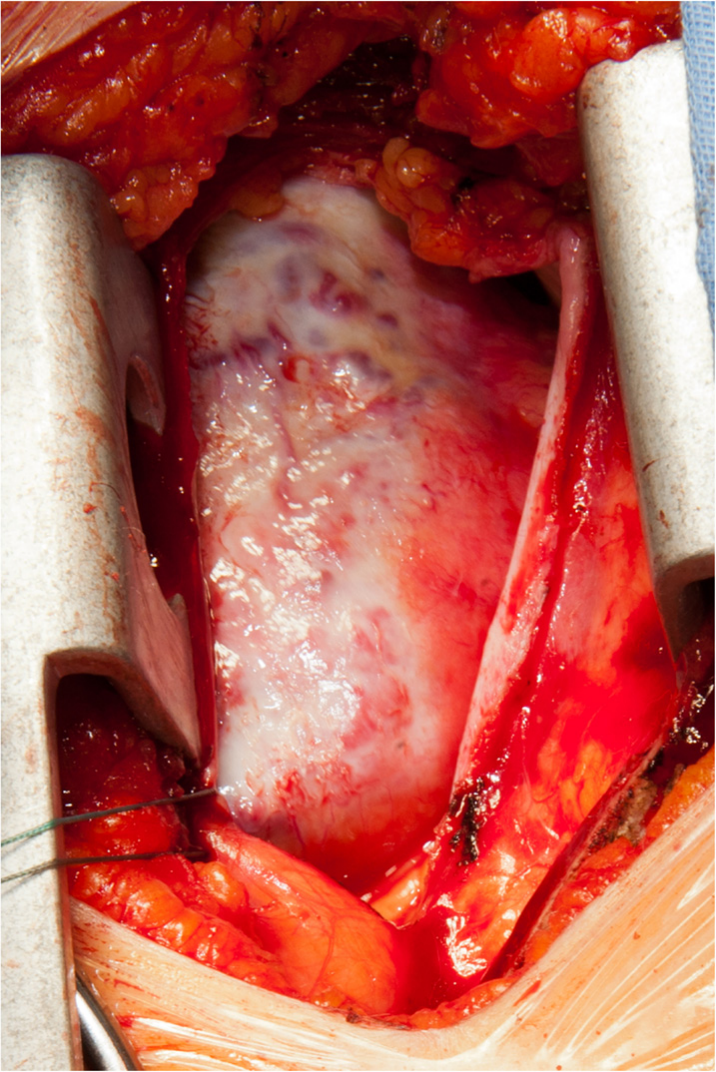
Myocardial biopsy through partial inferior sternotomy

**Fig. 5:**
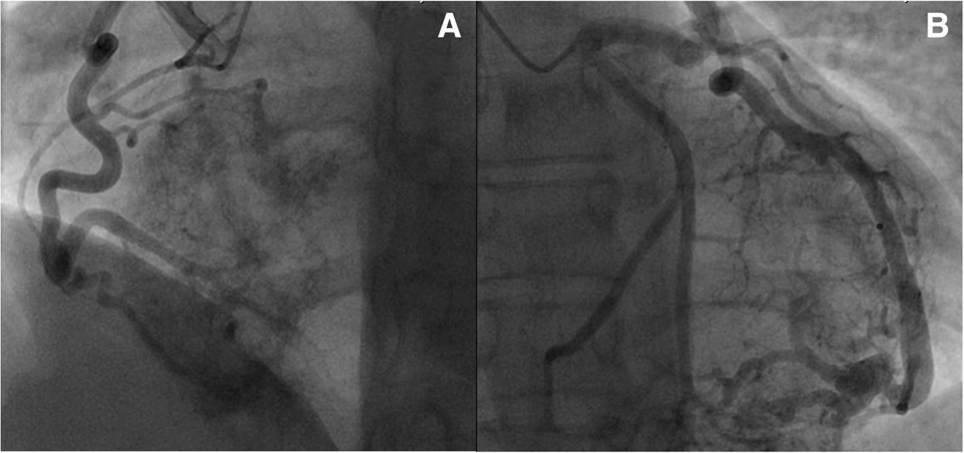
Left circumflex artery (**b**) and right coronary artery (**a**) with connection to the tumor showing a characteristic tumor blush

**Fig. 6:**
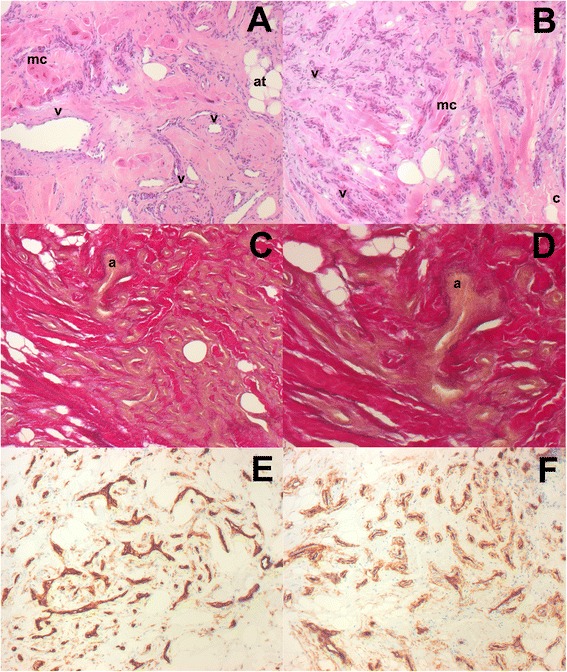
Hematoxylin and eosin stain (**a** and **b**, magnification 100×) showing the intracardiac angioma consisting of vessels (*v*), adipose tissue (*at*), collagen (*c*) and myocardial cells (*mc*). Elastic van Gieson stain (**c**, magnification 100× and **d**, magnification 200×) confirms the presence of arterioles (*a*) in the tumor. Strong positivity for CD31 (**e**, magnification 100×) and CD34 (**f**, magnification 100×) supported the vascular origin of this tumor

A transvenous right ventricular biopsy was performed to gain tissue material for histological analysis; however, the obtained material was insufficient for a definitive diagnosis. Our patient was then referred for an open myocardial biopsy via a partial inferior sternotomy (Fig. 4). Previously, a coronary angiography was performed to identify the feeding arteries of the large tumor. Coronary angiography revealed ectatic coronary arteries without any stenosis. A biventricular tumor was visualized on angiography with connection to both her right coronary artery and her left circumflex artery, showing a characteristic tumor blush [3] (Fig. 5). Histopathological analysis led to the diagnosis of a benign vascular tumor. Examination of the tumor revealed numerous capillaries, arterioles, and venules embedded in a collagen-rich matrix (Fig. 6a–d). Some vital heart muscle cells within the tumor mass could also be observed (Fig. 6a, b). Cells showed strong positive staining with antibodies against CD31 and CD34, which supported the vascular origin of this tumor (Fig. 6e, f). Histological findings were consistent with a benign intracardiac angioma. The first imaging follow-up was performed 2 weeks later by CMR, and showed an unchanged result. Because our patient was asymptomatic without signs of heart failure or arrhythmia, and the large tumor proved to be surgically unresectable, we decided on conservative management with regular clinical and imaging controls. Nine months later our patient presented to our department for her regular follow-up appointment without any major complications. Echocardiography showed no significant progress of the tumor.

## Discussion and Conclusions

Primary cardiac neoplasms are rare and have an incidence range of 0.0017–0.28 % as reported in autopsy studies [1]. Only about 5 % of all benign cardiac tumors are angiomas [1, 2]. On histology, hemangiomas can be classified into cavernous type, capillary type, and arteriovenous type. The most common location for a cardiac hemangioma is the left ventricle, followed by the right atrium, right ventricle, and left atrium. Hemangiomas can present at any age in both genders [4].

We report the case of a 35-year-old Caucasian female patient with an incidental finding of a large intracardiac angioma that represented a challenge for diagnosis and therapy. Echocardiography and CMR imaging are the most important diagnostic modalities for the evaluation of intracardiac masses [5, 6]. In addition, coronary angiography is helpful for further characterization of the tumor (for example, the degree of vascularization and relationship to the coronary arteries). Our patient was evaluated extensively by all the methods mentioned above in order to make the best treatment decision.

There are very limited data with respect to the treatment of patients with these extremely rare benign intracardiac vascular tumors [7]. Total surgical excision of benign angiomas is not routinely undertaken because of the highly vascular nature of these tumors, unless there are severe symptoms like ventricular tachycardia or cardiac tamponade [2]. There are some reports describing severe complications like ventricular arrhythmias or sudden cardiac death [8]. Only a few surgical resections of right ventricular hemangiomas have been reported in the literature; most of them were symptomatic [9]. Treatment of an asymptomatic benign intracardiac angioma remains controversial, because the evolution of such lesions is unpredictable—it may be constant in size, but can proliferate and grow uncontrollably. Fan *et al*. recently reported the total surgical resection of an incidentally discovered large hemangioma attached to the left ventricle with good results after 20 months of follow-up [10]. Alternatively, other authors suggest conservative management, especially when the patient is asymptomatic, the tumor is not compressing any major structures, the patient is not hemodynamically compromised, and when surgical risk is high [4, 11]. For example, Gribaa *et al*. reported a case of a left-sided cardiac hemangioma in an adult woman that was managed conservatively for 11 years without major complications [11].

The choice between surgical and conservative management should be made on an individual basis for each patient. Malign arrhythmias, congestive heart failure, ventricular outflow tract obstruction, coronary insufficiency, and higher grade atrioventricular blocks are especially alarming symptoms that may promote a surgical approach. Interdisciplinary discussion involving the cardiologist, cardiac surgeon, and oncologist should be undertaken in such cases.

## Consent

Written informed consent was obtained from the patient for publication of this case report and any accompanying images. A copy of the written consent is available for review by the Editor-in-Chief of this journal.

## Additional files

Additional file 1: Short axis multislice on cardiovascular magnetic resonance imaging (AVI 35056 kb)

Additional file 2:  Three-chamber view on cardiovascular magnetic resonance imaging (AVI 30355 kb)

Additional file 3:  Perfusion in four-chamber view on cardiovascular magnetic resonance imaging (AVI 15915 kb)

## Abbreviations

CD31: cluster of differentiation 31, also known as platelet endothelial cell adhesion molecule
CD34: cluster of differentiation 34, also known as hematopoietic progenitor cell antigen
CMR: cardiac magnetic resonance (imaging)
